# Supplementary material for: Public welfare donation, rent sharing, and income gap within enterprises
Source: PLoS One. 2024 Sep 5;19(9):e0309387. doi: 10.1371/journal.pone.0309387 (PMC11376583; doi:10.1371/journal.pone.0309387)
Supplement: Attachment — Submitted filename: Response to Reviewers.docx [file pone.0309387.s001.docx]

Dear Editor and Reviewers,

We would like to appreciate your effort for having reviewed our submitted manuscript and giving us so many valuable suggestions and helpful comments for improving the manuscript.

In the revised manuscript, we have improved this paper thoroughly according to your suggestions and comments. **And we marked the revised parts in red font**.

We are highly appreciated your great support and kind help in improving the quality of this paper. Our detailed responses to all of your individual comments are described in the following parts.

1. I have problems to understand what is meant due to incorrect English, such as CSMAR database should be “China Stock Market Accounting Research Database”. The authors should definitely employ a language editor before the paper gets published.

Response 1: Thank you for your valuable suggestions. We have hired a language editor to make compliance modifications and edits to the paper.
2. There is the logical contradiction between the first paragraph of “Theoretical analysis and research hypothesis” with the subsequent arguments, such as “when the public welfare donation promotes the improvement of the business performance of the enterprise, employees will share the rent of the enterprise”, especially the paper fails to distinguish the concepts of managers and employees in the first paragraph of “Theoretical analysis and research hypothesis” clearly.

Response 2：This article provides a detailed explanation of the concepts of management and ordinary employees in the dependent variables (1) and (2). In order to provide readers with an understanding of these two concepts when reading the first paragraph, an explanation has been provided in footnote 1 of the first paragraph.

When public welfare donations promote the improvement of business performance, employees will share the rent of the company. "The term" employees "refers to all employees of the company, including management and ordinary employees.
3. Some explanatory variables in the table are labeled incorrectly(the column (4) in table 5, Table 8).

Response 3： Thank you for discovering and pointing out the issue. We have made modifications to the errors in Tables 5 and 8.
4. the paper should explain the reason of using the fixed effect model to solve what kind of endogenous problem. And the paper needs to verify the applicability of the fixed effects model through Hausman test.

Response 4：Due to the presence of certain factors in the model that may not change over time or individuals, these factors are difficult to observe and may be related to disturbance terms, ultimately leading to endogeneity issues. So in order to control for the impact of these factors, we adopted a fixed effects test to alleviate the endogeneity problem of missing variables in the model. In ad dition, this article also validated the applicability of the fixed effects model through the Hausman test, and the test results are listed in the last row of Table 6.
5. The article requires a theoretical analysis of the reasons for the establishment of tax and performance mechanisms.

Response 5： The reasons for tax and performance mechanisms have been added to the text. According to the Production Supply Theory, taxation is a costly expenditure for enterprises. Tax incentives can reduce the operating costs of enterprises, keep the output level unchanged while reducing production inputs, and improve the production efficiency of enterprises. According to the theory of economic growth, as operating costs decrease, enterprises can expand their R&D investment, improve their innovation capabilities, and thereby promote the improvement of enterprise performance. Moreover, tax preferential policies can also guide enterprises to invest resources in more efficient areas, which can also promote the improvement of enterprise performance (Tang Hongxiang et al., 2020). Chinese scholars have made a lot of research results on the impact of tax incentives on corporate performance. The empirical results show that tax incentives have a positive impact on corporate technological innovation performance (Jia Jia, 2017), financial performance (Cai Chang et al., 2017), and investment performance (Yu Guansheng et al., 2023). The pre-tax deduction of public welfare donations, as an important tax preferential policy, can also play a role in increasing corporate performance.
