# Supplementary material for: Public welfare donation, rent sharing, and income gap within enterprises
Source: PLoS One. 2024 Sep 5;19(9):e0309387. doi: 10.1371/journal.pone.0309387 (PMC11376583; doi:10.1371/journal.pone.0309387)
Supplement: Attachment — Submitted filename: Response to Reviewers2.docx [file pone.0309387.s002.docx]

Dear Editor and Reviewers,

We would like to appreciate your effort for having reviewed our submitted manuscript and giving us so many valuable suggestions and helpful comments for improving the manuscript.

In the revised manuscript, we have improved this paper thoroughly according to your suggestions and comments. **And we marked the revised parts in red font**.

We are highly appreciated your great support and kind help in improving the quality of this paper. Our detailed responses to all of your individual comments are described in the following parts.

1. Corporate charitable donation is essentially a way of benefit sharing, and the income distribution gap between management and employees is also a manifestation of corporate social responsibility. Therefore, to some extent, this article discusses the relationship between the fulfillment of social responsibility within and outside the enterprise, or the allocation of economic resources between external and internal responsibilities of the enterprise, The article should appropriately elaborate on the impact of this issue on the research process and findings from the perspective of different forms of social responsibility.

Response 1: Thank you for your valuable suggestion. Corporate social responsibility includes four aspects: economic responsibility, legal responsibility, ethical responsibility, and charitable responsibility. In the introduction of the article, we elaborated on the impact of charitable donations on different forms of social responsibility. In addition, we also emphasize that the corporate social responsibility discussed in this paper specifically refers to charitable responsibility.
2. The theoretical analysis section in the article involves a variety of theories: principal agent theory, effective wage theory, bargaining theory, The rent sharing theory, which are complicated and overloaded. authors should accurately and concisely use related theory to explain.

Response 2：Thank you for your valuable suggestion. This article mainly uses rent sharing theory and bargaining theory, with the specific idea that public welfare donations increase the rent of enterprises. According to the rent sharing theory, both the management and ordinary employees of enterprises require rent sharing. Then according to the bargaining theory, the bargaining power of ordinary employees is weak, while the bargaining power of management is strong, the amount of rent shared by the two will be different, which leads to an widening income gap within the enterprise. We have adjusted the first paragraph in the theoretical analysis and research hypothesis section of the article.

3. I think the paper would benefit from a strong discussion section to add to the current 'conclusions and policy' section that needs to tie everything together, with the extant literature, including why this theoretical approach was chosen, whether there are any conflicting theories that were considered, why the fixed effects approach was chosen over other statistical approaches, why these particular robustness or sensitivity tests were done and importantly explain the limitations of this statistical and theoretical approach. While some of these components are presented in the results and introduction it feels a little scattered and not cohesively tied to current literature. I especially could not find the limitations discussed anywhere.

Response 3: Thank you for your valuable suggestion.We have made changes to the suggestions you made in our conclusions and policies.

Consistent with the research of Kong, D. et al. (2017) [35], we controlled for industry and annual fixed effects in benchmark regression, thereby controlling for influencing factors that change over time or industry. In addition, to alleviate the impact of endogeneity issues on our observation results, Heckman test[43]and control for individual fixed effects[44]were used. This result also passed a series of robustness tests. However, despite conducting a series of robustness tests, we were unable to rule out the interference of all endogeneity issues (Card et al., 2016)

In terms of research limitations, we have added content on model design. Considering that the rental sharing theory (Zhou W., et al., 2014) and bargaining power theory (Auerbach,A., 2018) we used cannot derive high-order regression results, we did not use a similar inverted U-shaped model (Liu,C. and Gao, J., 2022). Future research can develop a U-shaped regression model of corporate public welfare donations on internal income inequality based on other theoretical deductions.

It should be explained here that we have already controlled for industry annual fixed effects in the benchmark regression, in order to maintain consistency with the core reference articles. To alleviate the interference of endogeneity issues on our observed conclusions, we further utilized individual fixed effects to alleviate endogeneity issues. In this section, we have also added corresponding literature to support and compare for discussion.
